# Gold as a Filling Material

**Published:** 1895-10

**Authors:** L. C. Moore

**Affiliations:** ’95 D. C. of M., Detroit, Mich.


### Gold as a Filling Material.

L. C. MOORE, '95 D. C. OF M., DETROIT, MICH.

Perhaps it would be well to speak of some of the properties of gold foil and of the means of producing it, for it must be of interest to those using this material to know of its manufacture. Ottolengue says Gold is pre-eminently the best material with which to fill teeth. The properities of Gold which adapt it for the restoration of carious teeth are its softness, enabling it to adapt itself to the form of the cavity; its tenacity which gives facility of introduction and of consolidation; its resistance of chemical action, which prevents waste of material by the various influences to which it is subjected; and its agreeableness of color, which when pure and unburnished, approaches more nearly the shade of the natural tooth than any other metallic substance. The writer is inclined to differ somewhat from the above opinion, given by Dr. Jack, of Philadelphia. As to the color, I am inclined to think that the gold color is least objectionable because custom and the relative value of gold over other precious metals have made it so. Being the best of metals, it is preferred by the majority of patients in locations where it is seen principally on account of its intrinsic value. It is safe to say that if other precious metals possessed the properties of gold and were equal in intrinsic value, they would be quite, if not more, popular as materials with which to fill teeth. So far as color is concerned, other silver colored metals are less conspicuous and, to my notion, would be preferable if they would retain their color as gold does. It is a fact that gold is the only precious metal of the yellow color. Its main disqualification is its great conductivity of heat, and it also unfortunately requires considerable time for its proper insertion, in addition to which it lacks some of the good qualities of other materials; but, nevertheless, it can be stated positively that the main reliance for the salvation of teeth which have decayed must be upon gold. There is no data sufficiently accurate to give evidence when gold was first used in dental surgery, but it was early seen to be far more durable and sightly than lead, which was the first material ever used as a stopping for carious teeth. The results from the use of gold as a stopping are satisfactory, and it is even found to succeed well when other materials have failed. It is furthermore voiced by the profession of all periods as the most satisfactory material in all respects with which to fill teeth, provided the position is not prohibitory and the quality of the tooth structure sufficiently dense to prevent the requisite amount of force without injury to the borders. These elements qualifying success are, it is true, various, as a tooth which is thought by some operators to be too soft to be packed with gold by his methods can be easily done with safety by another whose methods are different, but the filling will be in each case of the same solidity.

The purity of gold foil is an important factor and a description of the refining process may be interesting as well as instructive. For the following facts I am indebted to Dr. Jack in the "American System":

"Purity of gold imparts to the foil the extreme softness it should possess and gives it the facility of being easily adapted to the surfaces of the cavity. However solid a filling may appear to the eye and touch, if the gold used does not possess the property of plasticity, there is danger of imperfect adaptation at some point. To point out the superiority of gold foil of to-day, we will compare the present results with the analysis of thirty years ago. A test made at the United States' mint in 1884 by Dr. E. Townsend of 1000 parts of crystal gold gave .993-½ pure gold, and was at that time declared to be equal to the foil in general use, and superior to most. Pure gold is rarely obtained, and from the great care and expense required, its production is well nigh impossible. The removal of the smallest particles of other metals would be important if practicable, since any metallic combination with it impairs its ductility, and likewise causes it to harden under pressure more than if it were absolutely pure. The slightest particles of arsenic, antimony, tin and lead would be sufficient to affect its ductility. The impurities of crude gold, as mined, consists principally of silver (which is always present), of iron and of either iridium, platinum or palladium, and it sometimes contains lead. Gold may be adulterated by arsenic and bismuth. The usual process of purification at the mints (which is the source from which gold foil manufacturers derive their gold) is by quartation. The silver is removed by nitric acid and the gold left in the form of a dark powder. The gold is now from .995 to .998 pure. It is also sometimes precipitated by the action of boiling sulphuric acid which raises the fineness to .999. Even at this degree it is not sufficiently pure to produce the softest and most tenacious foil. Manufacturers are obliged to subject it to further processes. As gold bullion from the mints varies considerably, it has to be approximated by the process of deflagation, which consists in continued fluxion of the gold with potassium nitrate which has the power of oxidizing the baser metals, as zinc, lead, arsenic and copper. It is further subjected to the action of sulphur dropped into the boiling metal which combines with the silver, and of corrosive sublimate for the removal of the last traces of iron. It is at last very nearly pure. Dr. Jack goes on to describe the making of gold foil. He says: "The ingot of refined gold is rolled between steel rolls into very thin ribbons the thickness of tissue paper. It is then weighed and divided into pieces of appropriate sizes to produce any of the numbers, 4, 5, 6 and so on. The little squares to the number of 200 are taken up by wooden pliers and placed in alternate layers with sheets of vellum which mass is called a cutch. A case of strong parchment is placed over the cutch in both directions to enclose the whole in a secure manner. The mass is now beaten by means of short-handled hammers upon a block of wood which should extend to the earth to afford solidity.

Considerable skill is required to attain evenness and to produce a good quality of foil. When the sheets are drawn out by beating until they extend to the edge of the vellum, they are removed, one by one, into a larger cutch and the process completed. The packet is repeatedly folded or rolled to prevent the gold adhering to the surface of the vellum and to keep the mass somewhat loose, Now, when the proper thinness is attained, the sheets are taken out and cut into size by a sharp reed on a leather cushion. The sheets are then accurately weighed, when they are ready for annealing.

This is an important and delicate process, and may be done in several ways, such as a frame of platinum wire gauze with a spirit lamp under it or in the muffler of a furance. In any event, it is important that the heat be high enough to thoroughly soften the gold and do it evenly to every part of the sheet. The sheets are then put in the books so familiar to dentists."

It would seem that gold foil has certain distinct and easily recognized properties which have direct relation to its use as a filling material. These are cohesiveness, softness, hardness, tenacity and fragility.

Cohesiveness is that property of gold which causes one particle or layer to adhere to another when they are laid together and if brought into actual contact by pressure, they become permanently united. This property is always present, independent of the purity of the foil. It is argued that silver increases cohesiveness, but at the same time lessens the softness of the gold foil. In our daily use of foil, we can easily see the great value of this property, for surely it is the means of making our filling a solid, impenetrable mass through which, provided it is properly inserted, caries cannot take place. It is a dam through which bacteria and salivary secretions cannot pass. Cohesiveness manifests itself directly after it is finished in the annealing process in its manufacture; but if lost is capable of being restored by heating to near a red color over the spirit lamp. Exposure to the atmosphere and various other conditions overcome this property, but heat will renew it. (The duration of the condition is very short, for an hour's time will show change, and in the course of a day it will be entirely lost.) For the knowledge of the cohesive property of foil, we are indebted to Dr. Arthur, who, after a number of experiments, recognized and revealed it. It is truly said, that this made a "revolution in dental surgery," and while it has increased the field of dentistry and has been of incalculable benefit, it has also done a great deal of harm by being used when not applicable. This holds true, especially on the margins of the cavities. A wrong impression has arisen in the minds of some, that cohesive gold is hard gold on account of the defectiveness of much of it now in use. Perhaps one reason for this is that when it is pure and accordingly soft, it is capable of being condensed into a condition of absolute solidity, and would consequently appear less yielding. If it becomes contaminated with other metals as copper, platinum, palladium, zinc, etc., as previously stated, while still retaining its cohesiveness, it is much harder, thus becoming harder to manipulate. In such a state, it is not fit for filling teeth, except for surfacing cavities in the grinding surfaces where mastication is extremely severe.

Non-cohesive gold foil is a gold which is incapable of cohesion, even after being highly heated. In this respect, it is distinguished from cohesive which, by exposure, loses this characteristic property. This difference has been the means of producing many unfortunate stoppings. This kind of gold has often been mistaken for and called soft gold in contra-distinction to cohesive foil. While non-cohesive gold, even when not absolutely pure, is more yielding when used in a mass than the purest cohesive, it is quite important that it be as pure as possible, for then on account of its softness and extreme tenacity, its working qualities are eminently shown. In this condition it is capable of being adapted to the margins of the cavities by slight force.

It may be said that this variety of gold may be used advantageously for filling accessible cavities at the beginning, the common cement of proximate cavities, and for lining the labeal walls in the anterior teeth. Non-cohesive foil, when perfectly pure, is nearly as soft as tin, and is somewhat easier to manipulate, but differs very little in its working qualities from it.

Now comes the question, "In what form can we best manipulate gold?" Formerly, there were but three ways in which it was used; the rope, the pellet and the tape, the first named being the most common. At the present time there is a large number of forms used, so that a person is not confined to any one kind. The pellet and rope are very rarely used and have given way to the mat, the compact and loose block, the compact and loose cylinder, the ribbon, rolled gold of considerable thickness plated with platinum and crystal gold. The tape is made by folding any portion of a sheet over and over until the desired width and thickness is produced. Sometimes it is an advantage to use small portions of the tape. It is then cut transversely in small pieces, which are called mats. These are especially valuable when non-cohesive gold is used in small cavities.

The compact block is formed by folding a tape on itself a number of times which is done by siezing it in the pliers and making turns of any desired size, either square or narrow. These, of course, should be of non-cohesive gold, as a mass so compact of other gold would become unmanageable by the cohesion of the layers. These blocks are useful in starting large proximate cavities at the cervical wall especially. They are also very useful in simple crown cavities. This form of block has been wrongly styled a cylinder.

The loose block is composed of cohesive gold, and is generally made of what is called corrugated gold, this being used as the layers only touch at certain intervals. This variety of gold is used only for building up after a foundation has been established.

The compact cylinder is made by rolling a tape of non-cohesive gold upon a fine broach, commencing at the end of the tape and rolling until the desired size has been reached. The size varies according to the thickness and width of tape. Compact cylinders are recommended for stoppings in buccal and coronal surfaces.

The loose cylinder, from outside appearance, resembles the previous kind, but it is decidedly different. This form of gold can be made only by the manufacturers. They are composed of several sheets laid one upon the other and wrapped loosely upon a needle-like piece of steel. After this rod has been removed, they are cut into assorted sizes, and are usually made of corrugated cohesive or semi-cohesive gold. This kind of gold is best employed in commencing fillings in which event it is usually annealed.

Ribbon gold is formed of whole sheets, and in some cases, of two sheets of flat cohesive gold, folded like a tape three times. These ribbons are then cut crosswise with shears into little strips which after being annealed are ready for manipulation.

Platinum plated with gold was first introduced for the purpose of filling teeth exposed to view, but now it has another and quite a valuable use and that is on the masticating surfaces of teeth worn down by years of constant grinding. Another advantage claimed is the tint which the two metals impart which resembles more nearly the shade of many teeth than gold alone. It might be well to say that this kind of gold is not adapted for composing the body of fillings, and should be used only for surfacing as it requires more force with the mallet than is safe upon the tooth at the beginning of the filling.

Dr. A. J. Watts was the first one to present crystal gold, some thirty years ago. The filling material at first was rather imperfectly prepared, being, in some cases, a simple precipitate of gold which was treated with nitric acid, the acid being neutralized by amonia, the gold was found in a crystalline condition. Afterwards it was thoroughly heated on a slide in a muffle, when it was ready to be used. This kind of gold became very popular and was used extensively for a time, but gradually fell into disuse, the reason for this being because of the impurities in the gold and also owing to the hurried and careless manner of introducing and packing it. Later, it was manufactured by electrolysis and since that time has been used moderately. Great care must be exercised in using crystal gold to prevent condensation in the primary manipulations; the pieces should not be too large, that they may be dispersed in a thin layer over the surface slightly compressed and afterwards thoroughly condensed by rather small instruments before inserting another layer. With such precautions, excellent results are obtained. But, after all, I can see no advantages in it over ordinary foil.

In annealing gold foil there does not seem to be any special methods introduced, it being only necessary to heat it in a flame which does not contain sulphur or phosphorus. Cohesive gold, heated in this way, its property is restored and non-cohesive gold, its toughness and density returned without making it harsh.

When a cavity is filled with pieces of gold, the mass is retained by the form of the cavity; but, unless the pieces of gold are placed in correct relation, each to the other, and to the walls, or are perfectly united together, the mass may become separated and part escape from the cavity, thus disfiguring the appearance of the filling and ultimate injury to the tooth. A writer on this subject gives three methods by which gold may be packed to prevent this accident.

1st. By the mechanical arrangement of the layers.

2d. By incorporation of the layers.

3d. By cohesion of the layers.

The first is applied to non-cohesive gold; the second, to the combination of cohesive with non-cohesive, and the third to cohesive gold exclusively.

I will not describe the manipulation of gold foil further, as this paper is already too long. Gold may be packed by hand pressure or malleted by hand, automatic or electric devices.

Notwithstanding all our knowledge of gold, its properties, and its virtues, I am reminded almost daily that we must not expect too much from our operations, for we are all doomed to disappointments in our indiscriminate operations. We are to acquire the skill, the technique and to learn to discriminate, but these things can only be learned by experience successes and disappointments. In our enthusiasm and ambition we are likely to undertake the construction of temples of fame which afterwards and altogether too soon, are tumbling ruins. So much is dependent upon the close observance of the technique. Gold alone is not the only requisite necessary in the construction of these temples. When we consider that a cavity in the tooth prone to decay is to be hermetically sealed with a foreign material, and that material a metal, it is not a very easy thing to do, although seemingly a simple matter to excavate a cavity and fill with a stopping of gold, but to seal that cavity absolutely against further decay, besieged by enemies upon all sides is not an easy task.

